# Adapting a communication coaching intervention for obstetric sonographers delivering unexpected news: A qualitative study

**DOI:** 10.1177/1742271X221147860

**Published:** 2023-01-28

**Authors:** Essie Kaur, Jane Arezina, Louise Bryant, Kathryn I Pollak, Gill Harrison, Ruth Bender Atik, Jen Coates, Natasha K. Hardicre, Roxanne Sicklen, Karen Horwood, Teresa Lardner, Jon Arnold, Rebecca Wallace, Judith Johnson

**Affiliations:** 1 School of Psychology, University of Leeds, Leeds, UK; 2 Health Services Management Centre, University of Birmingham, Birmingham, UK; 3 Specialist Science Education Department (SSED), Leeds Institute of Cardiovascular and Metabolic Medicine, School of Medicine, University of Leeds, Leeds, UK; 4 Division of Psychological & Social Medicine, Leeds Institute of Health Sciences, University of Leeds, Leeds, UK; 5 Duke Cancer Institute, Duke University School of Medicine, Durham, NC, USA; 6 Society and College of Radiographers (SCoR), London, UK; 7 School of Health & Psychological Sciences, City, University of London, London, UK; 8 The Miscarriage Association, Wakefield, UK; 9 Sands, London, UK; 10 NHS England, Leeds, UK; 11 Barnet Hospital, Royal Free London NHS Foundation Trust, Barnet, UK; 12 Independent Lay Expert, Leeds, UK; 13 Fetal Anomaly Screening Programme, Public Health Commissioning and Operations, NHS England, UK; 14 Tiny Tickers, Leeds, UK; 15 Royal Gwent Hospital, Aneurin Bevan University Health Board, Newport, UK; 16 Bradford Institute for Health Research, Bradford, UK; 17 School of Public Health and Community Medicine, University of New South Wales, Sydney, NSW, Australia

**Keywords:** Communication, breaking bad news, ultrasound, burnout, pregnancy, workforce

## Abstract

**Introduction:**

Despite widespread recognition that communicating unexpected news during obstetric ultrasound examinations is challenging, there is a dearth of research investigating how to teach evidence-based communication to sonographers. Communication Coaching is a supportive, positive method that has previously been associated with improvements in communication, patient satisfaction, and reduced burnout in clinicians. However, to date, no study has coached sonographers. This study explored stakeholders’ views on a proposed Communication Coaching intervention and used these data to adapt the intervention for use with qualified obstetric sonographers.

**Methods:**

Semi-structured interviews were conducted with people who have a vested interest in unexpected news delivery and thematic analysis was conducted on the data. Eight sonographers, six people with lived experience of receiving unexpected news and six representatives from third-sector organisations who support expectant parents were recruited (18 women; 2 men, aged between 21 and 75 years).

**Results:**

Participants viewed the planned Communication Coaching intervention favourably and suggested adaptations. The two main themes were (1) the practicalities of coaching, and (2) content. The first theme had four subthemes: (a) brief and flexible structure, (b) online modality, (c) sensitive and positive coach and (d) organisational awareness. The second theme had three subthemes: (a) specific language and behaviour recommendations, (b) adaptable to different service-users and situations and (c) confer relevant emotional skills and techniques.

**Conclusions:**

Communication Coaching could be a feasible and acceptable intervention for qualified sonographers if specific, limited adaptations are made as recommended by the stakeholders. Further evaluation of the intervention in practice is necessary.

## Introduction

In the United Kingdom, ultrasound practitioners routinely diagnose miscarriage, intrauterine death, and physical conditions while undertaking obstetric ultrasound examinations and then deliver this news directly to expectant parents.^1,2^ Sonographers undertaking obstetric ultrasound examinations are required to communicate in ‘real-time’ without any time to gather or formulate their thoughts in private before they speak with expectant parents.^2^ Having to deliver unexpected news while also ensuring that they obtain the required information from the scan, combined with the potential emotional distress that expectant parents can feel in response, mean that sonographers often find these situations technically demanding and psychologically stressful.^3^ In a qualitative interview study with UK sonographers, participants described news delivery encounters as generating uncertainty in relation to both what findings may reveal and how expectant parents may react.^2^ Over time, some sonographers felt delivery of unexpected news could become a form of ‘repetitive strain injury’, and an emotionally draining part of their role.^2^ These findings have been echoed by research studies internationally, with ultrasound practitioners reporting that they struggle to know what to say and how to manage expectant parents’ emotional reactions to unexpected news.^4^ In the present report, the term ‘sonographers’ will be used to refer to all practitioners who undertake obstetric ultrasound scans. The term ‘unexpected news’ will be used in place of ‘bad news’ or other similar phrases, due to its greater relevance in obstetric ultrasound and the preferences of expectant parents.^5^

Despite widespread recognition that it is challenging to communicate unexpected news in obstetric ultrasound, there has been a dearth of research investigating how best to teach effective communication skills to sonographers. In a study of UK sonographers, it was found that the majority had received post-qualification training in the delivery of unexpected news but that this often had been completed in their own time and at their own expense.^6^ Furthermore, while sonographers expressed a desire to learn about how to deliver such news via real clinical encounters, most training approaches relied on lectures and group discussions.^6^ Given current high levels of sonographer stress and burnout, identifying suitable ways to support sonographers with the potentially more stressful aspects of their work is important and timely.^7^

While few studies have investigated interventions for unexpected news delivery in sonographers, there is a wider literature testing such interventions in other clinicians.^8^ These studies have indicated that such training is effective, resulting in large improvements in communication skills.^8^ The effectiveness of training is further improved when interventions use a news delivery framework, such as the ‘SPIKES’ protocol.^9^ The ‘SPIKES’ acronym represents the recommended stages in news delivery for physicians, namely ‘Setting up the interview’, ‘assessing the patient’s Perception’, ‘obtaining the patient’s Invitation’, ‘giving Knowledge and information to the patient’, ‘addressing the patient’s Emotions’ and ‘Strategy and summary’.^10^ SPIKES is the most widely used news delivery framework in medicine, but it assumes that physicians will have time to formulate their words prior to speaking with patients, which is the first step in the framework (‘Setting up the interview’).^10^ For this reason, SPIKES is inadequate for obstetric sonographers, who communicate potentially ambiguous findings to expectant parents without time to prepare before speaking. In response to this gap, UK consensus guidelines for the delivery of unexpected news in obstetric ultrasound: The ASCKS framework was devised by the Improving News Delivery in Ultrasound (INDIRA) writing group, which is specifically tailored for obstetric sonographers.^5^ ‘ASCKS’ stands for ‘Avoid assumptions and loaded words’, ‘Set up the scan’, ‘Clear, honest information’, ‘Kindness and compassion are key’ and ‘Self-care’ and was generated via a consensus approach involving sonographers, other stakeholders and people with lived experience of receiving unexpected news.^5^

Most methods to train clinicians have involved workshops and simulated patient encounters, which is contrary to the communication training preferences previously expressed by sonographers, who emphasise a preference for learning from real clinical encounters.^8^ However, one intervention, ‘Communication Coaching’, trains clinicians using transcripts of their own patient encounters and as such, aligns more closely with these preferences.^6,11^ Communication Coaching is a one-to-one approach involving three 30- to 45-minute sessions between the coach and clinician.^12^ The first session is an introduction to the communication principles, and the second two sessions use transcripts of the clinician’s own audio recorded patient consultations to coach them.^12^ The coaching approach draws on adult learning principles whereby strengths are highlighted and possible ‘tweaks’ are suggested.^11,13^ Communication Coaching is a flexible and positive approach, designed to help clinicians to communicate more empathically by building their confidence and skillset.^11^

Previous studies indicate that Communication Coaching is feasible and acceptable to clinicians and is associated with higher quality communication and lower burnout.^14–16^ However, studies have previously been limited to clinicians based in the United States, and it is unclear how the intervention would need to be adapted to make it accessible for sonographers and relevant for their care recipients within other healthcare systems. To address this, the current study aimed to adapt the Communication Coaching intervention for qualified UK sonographers working within the National Health Service (NHS). Adapting interventions is more efficient and less resource intensive than creating ones *de novo*.^17^ It is recommended when the setting where an intervention will be delivered is significantly different to the context it was initially created for.^17^ There were two main research objectives:

1. To explore the views of stakeholders on a proposed communication coaching intervention
2. To use this data to adapt the communication coaching intervention for use with qualified obstetric sonographers

## Methods

### Design and analysis

Semi-structured interviews were conducted and data was analysed using thematic analysis.^18^

### Ethical approval

Ethical approval was granted by the School of Psychology Ethics Committee at University of Leeds (Approval number: 220, approval date: 10/3/2021).

### Sample

We interviewed 20 participants, 18 women and two men, aged between 21 and 75 years. Purposive sampling was used to select a variety of participants from three groups within the United Kingdom; qualified obstetric sonographers, those with lived experience of receiving unexpected news during pregnancy which was identified via ultrasound, and representatives from third-sector organisations who work with expectant parent groups. Eight sonographers, six participants with lived experience and six representatives from third-sector organisations were recruited. Nine participants were from Southern England, five were from Northern England, five were from the Midlands and one was from Scotland. All participants were UK-based residents. Twenty interviews provided enough information power for a thorough thematic analysis because of the narrow and novel nature of the research topic.^19^

### Recruitment

We recruited via Twitter and Facebook between July and September 2021. Tweets and posts detailed the research background, participant requirements, and the research team’s contacts details.

### Data collection

Semi-structured interviews were conducted via Microsoft Teams or Zoom. Twenty 30- to 60-minute interviews were conducted. Informed consent was verbally captured using the digital platform’s screen recording and transcription functions. To gauge the feasibility and adaptations required for the proposed intervention, participants were asked to read the intervention plan beforehand. The interview schedule was used in a flexible and conversational manner to ask interviewees a series of questions relative to the proposed coaching intervention, including prompts and follow up questions (Table 1). Following the interview, demographics were collected, and a study brief was delivered.

**Table 1.** List of questions and prompts used for the interview schedule.

1. What led you to be interested in the communication of unexpected news in obstetric ultrasound? 2. What was your initial reaction to the planned intervention? [Reference to the document and check that the participant has read this]. a. Is there anything you see as positive or potentially problematic? b. Did you have any questions or was there anything on it you were unsure about? 3. What did you think of the intervention structure? a. Number/length of sessions. b. When/what time should sessions be held. c. How far in advance should they be scheduled? d. What length of time should there be between coaching sessions? 4. What were your thoughts on the coaching approach? a. Audio-recordings appropriate? b. How do you think audio-recording *and/or* transcriptions of sessions will be received by sonographers? c. Sonographers will be asked to read the Improving News Delivery in Ultrasound (INDIRA) guidelines *or* summary paper in between the first and second session, what are your thoughts on this? d. Should clinic type be stipulated or flexible (e.g. early pregnancy and assessment unit vs. foetal anomaly screening programme)? e. Concerns around consenting patients? 5. What were your thoughts on the target communication behaviours? a. Were the *general communication behaviours* all relevant? b. Are some communication behaviours missing? or should some be removed? c. How closely should ultrasound-specific target communication behaviours be discussed and focused upon? d. How do you think these can be best presented to sonographers? (e.g. use of tables/worksheets) 6. What is your view on completing the coaching over a video platform (e.g. teams, zoom)? a. How do you think sonographers will react to this? b. Is there anything that could be put in place to make this as engaging as possible? c. Are there any ethical issues which should be considered here? 7. What did you think of the assessment approach? a. Do you think sonographers will be comfortable with having their communication practice assessed? b. Which communication practices in particular do you think should be focused on by the assessment? (e.g. empathy, ultrasound set-up, use of neutral language, etc.) c. Is there anything which should be assessed which our plan is currently missing? 8. Thank you for sharing your thoughts with us. Is there anything else you would like to comment on?

### Data analysis

Thematic analysis was applied to the dataset.^18^ Prior to analysis, all transcripts were anonymised, accuracy checked against the original recordings and a first read of all transcripts was done to establish familiarity with the data. Two researchers (E.K. and J.J.) independently analysed and openly coded an initial subset of transcripts. Initial codes were assigned according to their characteristics and formalised into emergent themes of ideas, opinions or suggestions identified by the coders. For inter-rater validity, the initial codes were compared and critically discussed between the research team. Codes were conceptualised to reflect the Communication Coaching intervention’s feasibility and necessary adaptations for sonographer suitability (see Table 1). Concepts were then categorised and ordered into a comprehensive framework of themes and sub-themes that reflected the research aims and questions. The coding framework was iteratively refined between the team through application to the next subset of transcripts. New codes were added as additional themes were identified, existing codes were rearranged and refined to best fit or represent the data and some codes were removed. The final coding framework was then applied to the remaining set of transcripts. Representative quotes were selected for each theme and sub-theme.

## Results

Recommended adaptations to the coaching intervention were grouped into two main themes: (1) the practicalities of coaching, and (2) content. The practicalities of coaching theme had four subthemes: (a) *brief and flexible structure*, (b) *online modality*, (c) *sensitive and positive coach* and (d) *organisational awareness*. The content theme had 3 subthemes: (a) s*pecific language and behaviour recommendations*, (b) *adaptable to different service-users and situations*, and (c) *confer relevant emotional skills and techniques* (see Table 2). All interviewees approved of the proposed intervention aims and components. Sonographers felt the Communication Coaching approach and the INDIRA guidelines could be beneficial for helping them develop a structured approach to delivering unexpected news in real-time.

*I think because there isn’t much teaching on it . . . we don’t get lectures at university. How to do it? There is no set way so it is an area that I think a lot of people struggle with in the day-to-day job.* (*p7; sonographer*)

**Table 2.** Themes and sub-themes with participant quotes.

Themes	Sub-themes	Key concepts	Participant quotes
1. Practicalities of delivering coaching	Brief and flexible structure	• Time pressures require brief, flexibly timed sessions.	“I honestly think if it’s not flexible it won’t work. I think sonographers, we work based on lists. So unfortunately, we don’t have the flexibility of changing our lists around because it takes a long time for admin staff to book everything. And in terms of admin, we all have different admin times, so you know if you give me a time, it wouldn’t work for someone else.”–p1 sonographer
	Online modality	• Sonographers need accessible learning resources. • Online delivery is favoured.	“Obviously, we’ve had a huge change in the way that training is delivered over the last 18 months or so, because face-to-face training is just not feasible, and I know that in a way that is an extension of the way that a lot of NHS type settings are working, that most training now is an e-learning module or people access that in their own time”. – p11; third sector organisation representative
	Sensitive and positive coach	Coach needs to be: • Warm, sensitive, positive, encouraging. • Able to foster a collaborative approach. • Responsive and supportive to needs.	“I would imagine that these coaching sessions are going to run into semi therapy sessions and you’re going to uncover some nasty things that have happened to them in for some of them where they’ve had, you know, they’ve been in scans were horrible things have been discovered and they’ve had to deliver really terrible news and people have taken in a variety of ways.” – p8; participant with lived experience of receiving unexpected news
	Organisational awareness	Needs to fit with: • The department’s local climate and capacity. • Cultural and professional expectations and practice. • Service-user needs.	“Is it that an actual sonographer doesn’t want to participate in the research anymore for whatever reason? Or is it that their manager just isn’t freeing up the time? Because I try to have protected time for myself to do CPD. . .” – p9; sonographer “I just think if it is unexpected findings being communicated, the last thing people want to hear then is would you mind taking part in this research study. So, I think you have to sort of set it up before, so that’s not an extra thing they’re dealing with. If they are hearing unexpected news.” – p15; participant with lived experience of receiving unexpected news
2. Content	Provide specific language and behaviour recommendations	Coaching needs to clarify the specific recommendations of the news delivery interaction including: • Verbal behaviours (words, phrases). • Non-verbal behaviours/ body language (eye-contact, use of touch). • Using Equipment and/or environment to aid communication.	“When I was being given bad news the sonographer. . . was rubbing my belly and I found that really strange. . . That made me quite uncomfortable.” – p4; participant with lived experience of receiving unexpected news
	Adaptable to different service-users and situations	• Accommodate different circumstances/events. • Sensitivity (cultural, emotional, psychological). • Individual differences (experience, capacity, wellbeing; willingness). • Setting, clinic type and physical environment.	“It’s about the variation of response from parents and which will be good for their learning experience is how they followed through any cases where unexpected news has been given.”–p6; third sector organisation representative “You adapt your communication to the person that you have in your room and you adapt that.” – p7; sonographer
	Confer relevant emotional skills and techniques	• Enable sonographers to practice managing their own and patients’ emotions and/or responses during communication.	“And in the fact that there’s not a lot of studies that recognize that the amount of emotion, emotional sort of burden that sonographers carry when breaking bad news. I didn’t know that myself before I started this job” – P1; sonographer

NHS: National Health Service; CPD: Continuing Professional Development.

### Practicalities of delivering coaching

#### Brief and flexible structure

All sonographers expressed that the burden of the intervention should be as low as possible, should not interfere with sonographers’ workloads and should be tailored to their working schedules. Participants from all groups felt brief and pre-scheduled sessions would enable sonographers to better manage their participation and enhance their willingness to engage.

*I honestly think if it’s not flexible it won’t work. I think sonographers, we work based on lists. So unfortunately, we don’t have the flexibility of changing our lists around because it takes a long time for admin staff to book everything. And in terms of admin, we all have different admin times, so you know if you give me a time, it wouldn’t work for someone else.* (*p1; sonographer*)

The importance of brevity was also reflected by participants with lived experience of receiving unexpected news, who felt research activities should not cause their appointments to be longer than usual. Specifically, it was suggested that permission for audio recording and equipment management should be sought during the waiting time to avoid any delays in accessing ultrasound appointments.

#### Online modality

All participants confirmed that delivering the coaching online and offering online resources would support accessibility. Some participants highlighted that the Covid-19 pandemic has resulted in an increased acceptance of online training. Even though some parents and third sector organisation representatives mentioned that in-person interaction offered benefits, the majority others reasoned that digital delivery of the coaching and online resources would be easier to access because it would reduce time and physical space demands, reduce risk of Covid-19 transmission and accommodate inflexible workloads and/or shift patterns.

Some third sector representatives noted that digital literacy and additional needs of individuals should be considered in the design otherwise it may act as a barrier to accessibility. For example, any software used in the intervention should be widely available and participants should not be expected to develop new digital knowledge:

*Obviously, we’ve had a huge change in the way that training is delivered over the last 18 months or so, because face-to-face training is just not feasible, and I know that in a way that is an extension of the way that a lot of NHS type settings are working, that most training now is an e-learning module or people access that in their own time’. (p11; third sector organisation representative)*

#### Sensitive and positive coach

The communication of unexpected news was often described as an intense and emotional experience by both sonographers and participants with lived experience of receiving unexpected news. Some sonographers noted that they do not have the time to process how the delivery of unexpected news affects them personally, which can leave them experiencing a build-up of suppressed emotions. Due to this, many sonographers reported experiencing symptoms of burnout. Sonographers indicated that to speak about news delivery experiences would require a coach who is sensitive, compassionate, and able to make sonographers feel safe and supported. Common adjectives used by all participant groups referred to coach characteristics being important and instrumental to the engagement and learning of the sonographer and when talking about coach requirements included ‘warm’, ‘sensitive’, and ‘positive’:

*I would imagine that these coaching sessions are going to run into semi therapy sessions and you’re going to uncover some nasty things that have happened to them in for some of them where they’ve had, you know, they’ve been in scans where horrible things have been discovered and they’ve had to deliver really terrible news and people have taken in a variety of ways.* (*p8; participant with lived experience of receiving unexpected news*)

#### Organisational awareness

Sonographers made explicit reference to their supervisors and/or team lead being a crucial influence on their desire and commitment for professional development and training. Sonographers stated that having their supervisors’ support was a fundamental and determining factor in whether they would participate in the intervention. As such, it was suggested that it would be necessary for the intervention to appeal to senior staff and department leads and to align with local organisational culture:

*Is it that an actual sonographer doesn’t want to participate in the research anymore for whatever reason? Or is it that their manager just isn’t freeing up the time? Because I try to have protected time for myself to do CPD.* (*p9; sonographer*)

Participants with lived experience highlighted that those attending obstetric ultrasound appointments may be experiencing heightened emotions already. They suggested that expectant parents should be informed about the study before the scan but should not be burdened with too much focus on improving ‘unexpected news’ delivery to avoid unnecessary distress. It was suggested that it would be more appropriate to explain the wider aim of improving sonographer communication:

*I just think if it is unexpected findings being communicated, the last thing people want to hear then is would you mind taking part in this research study. So, I think you have to sort of set it up before, so that’s not an extra thing they’re dealing with. If they are hearing unexpected news.* (*p15; participant with lived experience of receiving unexpected news*)

### Content

#### Provide specific language and behaviour recommendations

Sonographers stated the coaching should clarify the specific recommendations of the news delivery interaction including verbal behaviours (e.g. words and phrases to use or avoid), non-verbal behaviours and body language (e.g. eye-contact, use of touch). Each noted there was no established ‘benchmark’ of language and behaviour recommended in their training, which incited uncertainty on what was perceived as an appropriate approach to delivering unexpected news. Sonographers believed this added significant pressure when having to formulate and deliver news in real-time and had a direct impact of their wellbeing. Participants with lived experience confirmed these sentiments, drawing on experiences where they felt the sonographers did not communicate the news effectively and appropriately:

*When I was being given bad news the sonographer. . . was rubbing my belly and I found that really strange. . . That made me quite uncomfortable*’. (*p4; participant with lived experience of receiving unexpected news)*

The physical environment was commonly referred to by participants with lived experience as an important factor in their overall experience. Some sonographers also expressed uncertainty regarding the use and placement of ultrasound equipment when preparing to deliver unexpected news in real time.

#### Adaptable to different service-users and situations

Sonographers wanted coaching practices and materials to address the wide range of findings they communicate during obstetric ultrasound scans. They also said that recommendations made during coaching should be adaptable to variations in the physical environment. All felt sensitivity should be embedded within all components of the intervention content. Respect for both sonographer’s and service-user’s cultural differences, emotional and psychological experiences during and in response to the delivery of unexpected news was also paramount. Most believed previous experience, perceived capacity, wellbeing and willingness should be accounted for on a person-by-person basis:

*It’s about the variation of response from parents and which will be good for their learning experience is how they followed through any cases where unexpected news has been given. (p6; third sector organisation representative)*

*You adapt your communication to the person that you have in your room. (p7; sonographer)*

#### Confer relevant emotional skills and techniques

All participants highlighted the emotional nature of unexpected news delivery encounters. Participants with lived experience of this detailed how and what shaped their experience, including their anxious thoughts, feelings of anticipation beforehand, prior experiences of pregnancy and interactions with the sonographer. Many described the sonographer as having the most influence in shaping this encounter. Recognising this, some participants suggested the coaching content should provide sonographers with skills in how to respond and manage distress.

*And in the fact that there’s not a lot of studies that recognize that the amount of emotion, emotional sort of burden that sonographers carry when breaking bad news. I didn’t know that myself before I started this job. (p1; sonographer)*

### Summary of findings

Communication Coaching was viewed favourably by stakeholders as a potentially useful intervention for supporting qualified sonographers with delivering unexpected news. Suggestions for how the intervention could be adapted to enhance feasibility, uptake and effectiveness were specific and actionable. Participants emphasised the working pressures which sonographers and ultrasound departments are currently facing and suggested that the intervention design would need to recognise and respond to this. Participants felt that this could be achieved by offering sessions flexibly and online. They also highlighted the benefits of working with senior staff to gain practical support for delivering the intervention. Participants discussed the emotionally sensitive nature of unexpected news delivery discussions and suggested that the intervention would need to be attentive to this by ensuring that coaches are trained to be compassionate and positive. One important point raised was the salience of language and behaviours in news delivery communications; participants said that suggestions provided in the coaching should be specific and evidence-based. Views of sonographer participants aligned with those of participants with lived experience and participants from third-sector organisations who work with expectant parent groups.

This information was then used to guide adaptations to the Communication Coaching intervention for sonographers (Table 3). The intervention was adapted with qualified sonographers in mind, rather than trainee sonographers, although it possible that with further adjustments, it could also be suitable in that group. All key principles of the original Communication Coaching intervention were retained, with some adaptations made to increase suitability for sonographers delivering unexpected news in obstetric ultrasound, such as the inclusion of specific language and behavioural recommendations based on the ASCKS framework.

**Table 3.** Key features of the adapted intervention: Communication Coaching for sonographers.

Features		How this relates to the original intervention
Structure	• One introductory session. • Two coaching-focused sessions. • All sessions to be flexibly delivered according to participants’ schedules.	Communication Coaching is an inherently flexible approach, but Communication Coaching for sonographers emphasises this more strongly; recognising current staff shortages in obstetric ultrasound departments and the lack of ring-fenced continuing professional development time sonographers receive.
Modality	• Online	Communication Coaching originally adopted an in-person format but has since evolved to use both in-person and online modalities. Communication Coaching for sonographers will exclusively use an online modality to ensure optimum flexibility for participants.
Approach	• Uses adult learning principles of positive reinforcement. • Behaviour change recommendations are ‘tweak’-focused.	This aligns to the original Communication Coaching intervention design. Criticisms are avoided entirely; strengths are reinforced and praised and suggestions for improvement are offered.
Focus	• Scans involving the delivery of unexpected news.	Previous Communication Coaching interventions have mainly been targeted at physicians and their specific focus has varied. The focus of Communication Coaching for Sonographers is unique but aligns to the original goal of Communication Coaching, which is to support clinicians with their most challenging encounters.
Content	• Coaching focuses on the ASCKS framework which provides recommendations for sonographers delivering unexpected news in obstetric ultrasound. • Coaching also aims to generally encourage empathic communication behaviours, such as naming the emotion expectant parents seem to be experiencing and offering wish statements, e.g. ‘This must be upsetting news. I wish things were different’.	The original Communication Coaching approach draws on the WISER framework which is designed for physician consultations (Walk in, sit down and make eye contact; Invite patient’s story with open-ended questions; *S*ay back what you’ve heard; Empathise; Revisit concerns).^16^ Communication Coaching for Sonographers shares similar underlying principles but utilises a tailored news delivery framework for sonographers, instead. As with the original Communication Coaching intervention, this framework is applied flexibly, as a tool to highlight the strengths that the sonographer shows in each encounter, recognising that different circumstances will require a variation in the specific words, phrases and behaviours a sonographer adopts. Tweaks are also suggested in a flexible manner depending on the tweak opportunities the coach identifies as being present within each exchange between the sonographer and service-user.
Materials	• ASCKS pocket card outlining the ASCKS framework and recommendations. • Audio-recordings of scans involving unexpected news delivery. • Summarised feedback on transcripts. • Annotated transcripts with highlighted strengths and suggested tweaks.	The materials align with those outlined in the original communication coaching interventions, with the substitution of the ASCKS framework in place of the WISER framework. Adaptation was made in the gaining of the audio-recordings. This involves ensuring that service-users are provided with information about the potential for recording prior to their scan, so they have time to consider this beforehand and the discussion does not extend the length of scans. A further adaptation involves the content of the recordings. In the original Communication Coaching intervention, clinicians record until they have a recording of a challenging patient encounter they would like to reflect on in coaching. As Communication Coaching for Sonographers focuses on scans involving unexpected news delivery, sonographers will record their scans until a scan involving the delivery of unexpected news occurs which they would like to reflect upon in coaching. As with the original Communication Coaching intervention, all recordings 1. Start at the beginning of the service-user encounter, 2. Run until the service-user leaves the room, 3. Are anonymized during the transcription process and 4. Are destroyed once the coaching session has been delivered (or as soon as possible after this within the context of an ethically-approved research project).
Coach attributes	• Positive and supportive. • Sensitive and compassionate.	While these attributes align to those outlined in the original Communication Coaching intervention, a particular emphasis on sensitivity is necessary for coaching in obstetric ultrasound due to the heightened emotion often involved in these encounters.

## Discussion

This is the first study to explore the adaptation of a communication training intervention for sonographers. A broad literature has explored the experiences of expectant parents when receiving unexpected news about a pregnancy which has been identified via ultrasound and has identified ways in which these experiences could be improved.^4^ Increasingly, literature has also begun to explore news delivery experiences of ultrasound practitioners and sonographers and has identified that sonographers feel unsupported with this task.^1–4,20^ Indeed, while several studies have now evaluated news delivery interventions in physicians,^8^ no research has yet tested the effectiveness of a news delivery intervention in sonographers. While post-qualification communication training is available to many sonographers, this is usually accessed independently, in sonographers’ own time and at their own expense.^6^ At present, there is no intervention which is embedded in services and accessible during sonographers’ working hours. Furthermore, there is no communication training intervention that draws on sonographers’ clinical experiences, in line with their expressed preferences for news delivery interventions.^3^ The present study addresses these gaps by producing recommendations for a Communication Coaching intervention which meets the needs of both sonographers and expectant parents, and which can be embedded within existing services.

In addition to improving sonographers’ communication practices, Communication Coaching is designed to be a positive, encouraging and supportive experience which strengthens participant confidence.^11^ When delivered to physicians, it was associated with reductions in burnout.^16^ Supportive interventions with the potential to reduce burnout in sonographers are currently crucial. Rates of burnout in sonographers were high prior to the pandemic, with a 2019 study of 90 UK sonographers indicating that over 80% of respondents were experiencing burnout.^6^ However, the Covid-19 pandemic has exacerbated existing stressors and introduced new ones, including fears of Covid-19 infection due to working on the front-line of healthcare and the physical discomfort of needing to wear personal protective equipment (PPE).^21,22^ In an Australian study, it was found that 41% of sonographers surveyed felt more isolated than usual during the pandemic, and that 78% were afraid of passing Covid-19 on to their family.^21,22^ A recent UK study found that over 90% of the sonographers surveyed met criteria for burnout.^7^ As such, there is a significant need for feasible, acceptable interventions which can be delivered to provide support for sonographers. The present study indicates that Communication Coaching could be a feasible intervention for organisations to consider.

The previous applications of Communication Coaching have been varied, and it has been used to enhance physician skills in motivational interviewing^16,23,24^ goals of care conversations^15^ and empathic communication.^12^ However, this is the first time that Communication Coaching has been adapted for a clinician group outside of the United States. Conducting research to adapt interventions is considered more efficient than creating de novo interventions and is recommended when the transferability of an intervention cannot be assumed.^17^ Qualitative research is frequently used to support adaptations^25–27^ and it is recommended that all relevant stakeholder groups are included to help triangulate findings.^25^ However, to understand whether an intervention has been successfully adapted for a population, feasibility studies are necessary.^26^ As such, the present study can be viewed as a springboard for future studies testing the feasibility and acceptability of Communication Coaching for Sonographers.

### Strengths and limitations

The study benefitted from the inclusion of participants from a range of stakeholder groups, which supported triangulation of results. It also benefitted from a strong research design involving in-depth one-to-one interviews which allowed for the generation of detailed data. It was limited by a gender imbalance in participants, with an under-representation of men, but this might be expected given that the ultrasound workforce includes a high proportion of women.

### Implications for research and practice

Communication Coaching was viewed positively by all groups of participants. Findings also suggested that all groups were aware of the time pressures sonographers and ultrasound departments are currently facing and that supportive interventions in this group will need to minimise time requirements and enhance flexibility of access. As such, the current results not only recommend the further testing of Communication Coaching for Sonographers, they also provide information which can be used by organisations looking to offer feasible and accessible supportive interventions for sonographers. In particular, interventions should be brief, flexible to sonographers’ schedules and delivered by professionals who are sensitive to the working context of sonographers.

## Conclusion

The delivery of unexpected news is challenging for obstetric sonographers and there is a lack of evidence-based, easily accessible interventions to support sonographers with this task. The current study presents findings which explore how Communication Coaching can be adapted for qualified obstetric sonographers. Results suggest that stakeholders viewed the possibility of delivering this intervention positively. The basic structure and approach of the intervention was considered acceptable and specific; practical changes which can be made to further enhance the feasibility of delivering this in sonographers were identified. Future research should evaluate the feasibility of delivering this intervention in practice.
